# Clinical and parasitological response to oral chloroquine and primaquine in uncomplicated human *Plasmodium knowlesi *infections

**DOI:** 10.1186/1475-2875-9-238

**Published:** 2010-08-19

**Authors:** Cyrus Daneshvar, Timothy ME Davis, Janet Cox-Singh, Mohammad Z Rafa'ee, Siti K Zakaria, Paul CS Divis, Balbir Singh

**Affiliations:** 1 Malaria Research Centre, Faculty of Medicine & Health Sciences, Universiti Malaysia Sarawak, 93150 Kuching, Sarawak, Malaysia; 2 University of Western Australia, Department of Medicine, Fremantle Hospital, PO Box 480, Fremantle, WA 6959, Australia; 3 Kapit Hospital, 96800 Kapit, Sarawak, Malaysia

## Abstract

**Background:**

*Plasmodium knowlesi *is a cause of symptomatic and potentially fatal infections in humans. There are no studies assessing the detailed parasitological response to treatment of knowlesi malaria infections in man and whether antimalarial resistance occurs.

**Methods:**

A prospective observational study of oral chloroquine and primaquine therapy was conducted in consecutive patients admitted to Kapit Hospital, Sarawak, Malaysian Borneo with PCR-confirmed single *P. knowlesi *infections. These patients were given oral chloroquine for three days, and at 24 hours oral primaquine was administered for two consecutive days, primarily as a gametocidal agent. Clinical and parasitological responses were recorded at 6-hourly intervals during the first 24 hours, daily until discharge and then weekly to day 28. Vivax malaria patients were studied as a comparator group.

**Results:**

Of 96 knowlesi malaria patients who met the study criteria, 73 were recruited to an assessment of the acute response to treatment and 60 completed follow-up over 28 days. On admission, the mean parasite stage distributions were 49.5%, 41.5%, 4.0% and 5.6% for early trophozoites, late trophozoites, schizonts and gametocytes respectively. The median fever clearance time was 26.5 [inter-quartile range 16-34] hours. The mean times to 50% (PCT_50_) and 90% (PCT_90_) parasite clearance were 3.1 (95% confidence intervals [CI] 2.8-3.4) hours and 10.3 (9.4-11.4) hours. These were more rapid than in a group of 23 patients with vivax malaria 6.3 (5.3-7.8) hours and 20.9 (17.6-25.9) hours; *P *= 0.02). It was difficult to assess the effect of primaquine on *P. knowlesi *parasites, due to the rapid anti-malarial properties of chloroquine and since primaquine was administered 24 hours after chloroquine. No *P. knowlesi *recrudescences or re-infections were detected by PCR.

**Conclusions:**

Chloroquine plus primaqine is an inexpensive and highly effective treatment for uncomplicated knowlesi malaria infections in humans and there is no evidence of drug resistance. Further studies using alternative anti-malarial drugs, including artemisinin derivatives, would be desirable to define optimal management strategies for *P. knowlesi*.

## Background

The simian malaria parasite *Plasmodium knowlesi *causes symptomatic infections in humans throughout South-east Asia in areas inhabited by its natural macaque hosts (*Macaca fascicularis *and *M. nemestrina*) and mosquito vectors of the *Anopheles leucosphyrus *group [1-8]. Early trophozoites of *P. knowlesi *are morphologically similar to *Plasmodium falciparum *and all the other stages resemble those of *Plasmodium malariae *[9]. However, unlike *P. malariae *infections, knowlesi infections can be severe and even fatal. A recent observational study found complications in 10% of patients and a 2% mortality [10].

Previous reports have indicated that patients with uncomplicated knowlesi malaria respond well to chloroquine treatment [1]. In one recent study, the mean parasite clearance time of 24 hours for knowlesi malaria patients was shorter than that for patients infected with *P. falciparum *or *Plasmodium vivax *[9]. There has, however, been no detailed description of clinical and parasitological responses following treatment and no previous assessment of antimalarial drug resistance. Such a study was, therefore, undertaken on patients with knowlesi and vivax malaria during hospital stay and on a weekly basis for a month.

## Methods

### Study site, design and patients

The present study was conducted in Kapit Hospital, which serves a population of 109,000 in Sarawak, Malaysia Borneo, an area with a reported annual malaria incidence of 1.2/1,000. A prospective observational study design was employed, using the WHO 28 day extended *in vivo *drug response assessment [11]. The patients were selected from a larger cohort participating in a pilot study and a subsequent observational assessment of consecutive malaria admissions between May 2006 and December 2007 [10]. Although there are currently no criteria for severity of infection in knowlesi infected patients, we have previously defined uncomplicated cases as those with a parasitaemia of ≤ 100,000/μL based on falciparum malaria data and the level of immunity in the community. In addition for the present study, patients that were selected i) had *P. knowlesi *or *P. vivax *monoinfections confirmed by PCR, ii) were at least 15 years of age, iii) were not pregnant, and iv) had no significant co-morbidities. Those who reported treatment with antimalarial drugs within the preceding 14 days were excluded. All patients gave written informed consent to participation. The study was approved by the Medical Research and Ethics Committee of the Malaysian Ministry of Health.

### Antimalarial therapy

All patients were given oral chloroquine 10 mg base/kg body weight, followed by 5 mg base/kg at 6, 24 and 48 hours (total dose 25 mg base/kg). At 24 hours, and after glucose-6-phosphate dehydrogenase deficiency had been excluded (SQMMR 720, R&D diagnostics, Holargos Greece), oral primaquine (15 mg base) was administered with food for either two consecutive days for knowlesi malaria (primarily as a gametocidal agent, as recommended by the Malaysian Ministry of Health for patients with *P. malariae*; there were no treatment guidelines for *P. knowlesi *at the time of the study and PCR confirmation was pending) or 14 days for vivax malaria (as also recommended by the Malaysian Ministry of Health). All drugs were administered by nursing staff on the medical ward where the patient was observed until parasite clearance (when two consecutive blood films 24 hours apart were negative for malaria parasites).

### Clinical and laboratory procedures

At recruitment, a detailed history and clinical examination were performed and recorded on standardized forms. Blood samples were taken for routine laboratory investigations including a full blood count, biochemical analyses, and blood cultures. In view of the possibility of cardio-respiratory complications developing during treatment [10], a baseline chest radiograph and 12 lead electrocardiogram were performed.

On admission and daily thereafter during hospitalisation, blood samples were collected on filter paper for subsequent DNA extraction and polymerase chain reaction (PCR) analyses to identify the species of *Plasmodium *and determine parasite clearance as described previously [1,9,12]. Blood was drawn for a full blood count at 0 and 24 hours. All patients had four-hourly monitoring of vital signs, daily clinical examination, daily blood sampling and blood films until parasite and fever clearance.

In randomly-selected sub-groups of patients with knowlesi or vivax malaria, additional samples were collected at three or six-hourly intervals for the first 24 hours to enable more detailed characterisation of initial parasite clearance. Because of the limited number of patients with *P. vivax *infections, twice the proportion was selected for this group compared to that in the *P. knowlesi *group.

Following discharge, patients were reviewed on days 7, 14, 21 and 28. Symptoms and clinical signs including the presence of splenomegaly or hepatomegaly were recorded, and further blood samples were taken, including blood films and filter paper samples for PCR analyses. If returning to Kapit Hospital for weekly visits was difficult, patients were asked to attend their nearest clinic or prepare their own blood spots on filter papers on days 7, 14 and 21, and to return on day 28 for full review. When patients did not attend a follow up appointment, efforts were made to contact them via telephone, through their employer if at a logging camp, through people from the same region who were seen at Kapit Hospital, or by use of the local radio station. Patients were excluded from the study if they missed a follow-up visit.

All blood films were examined by two microscopists who were blinded to all clinical and other data. Parasite counts were calculated from the number of parasites per 500 white cells, and a density calculated using the patient's own white cell count. Stages were classified as early trophozoites (ring forms and non-pigmented trophozoites), late trophozoites (pigmented parasites with two or less nuclei), schizonts (pigmented parasites with more than two nuclei), or gametocytes as previously described [9].

### Outcome measures

Fever clearance time (FCT) was defined as that from admission to when the axillary temperature reached, and remained below, 37.5°C for more than 48 hrs. The time to clearance of 50% and 90% of the admission parasitaemia (PCT_50 _and PCT_90 _respectively) were determined using least squares linear regression of log-transformed data. The time to a parasitaemia < 5 parasites/μL and the parasite reduction ratio at 24 hours were also calculated. Early treatment failure was defined as a history of fever on day 3, or the development of severe malaria on day 1 to 3, parasitaemia day 2 greater than on day 0, pyrexia ≥37.5 °C day 3, parasitaemia on day 3 ≥25% of day 0. Late treatment failure was defined as severe malaria after day 3 with associated parasitaemia or pyrexia ≥37.5 °C and parasitaemia present day 4 to day 28. These definitions are derived from the WHO criteria for low to moderate transmission areas [11]. Parasite DNA was extracted from the blood spots collected on filter papers daily and at follow-up and was analysed by nested PCR assays to determine the time to negative PCR and to assess for parasite recrudescences and re-infections [1,12].

### Statistical analysis

Data were analysed using SPSS software, version 14 (SPSS). Normally distributed variables were expressed as the mean and standard deviations and comparisons made using the Student *t*-test. Non-normally distributed variables were expressed as the median and interquartile ranges, and comparisons were made using the Mann-Whitney U test. Proportions were compared with the Fisher's Exact test. A P value < 0.05 was considered significant in all cases.

## Results

During the study period, 145 out of 187 patients admitted with malaria had single *P. knowlesi *or *P. vivax *malaria infections confirmed by microscopy and PCR. Of these, 111 (77%) patients had *P. knowlesi *and 34 (23%) had *P. vivax*. There were 82 patients with knowlesi malaria (73.9%) who were eligible for, and agreed to have, 28-day WHO follow-up and/or six-hourly sampling. Of these, 49 were enrolled for day 28 follow-up only, 24 for both day 28 follow-up and six-hourly sampling, and nine for six-hourly sampling only. All 33 patients (40%) who consented to six-hourly sampling completed the study but, of the 73 scheduled for 28-day *in vivo *assessment, 13 did not attend for at least one scheduled review and were excluded. In the vivax group, 29 (85%) of the 34 patients admitted met study criteria and 23 (79%) had six- hourly blood films taken during the first 24 hours. The majority of the vivax patients (65%) were logging-camp workers returning from Asia-Pacific countries with a higher incidence of malaria than the Kapit region. Because 14 (61%) patients left the study area to resume employment elsewhere, there were insufficient numbers for analysis of the WHO 28 day extended *in vivo *drug response.

Subject characteristics at study entry are summarized in Table 1. The patients in the vivax group were younger, all male and more likely to have had malaria previously. Of the patients in the knowlesi group, most were of Iban ethnicity and had been born in the Kapit division. All of the communities along the large rivers and tributaries were represented.

**Table 1:** Demographic features of patients at study entry

Variable	*P. knowlesi*	*P. vivax*	*P*-value
	(n = 73)	(n = 23)	
			
Age (years)	46.4 ± 13.42	38.5 ± 7.62	< 0.01
			
Male	58	100	< 0.01
			
Iban Ethnicity	96	83	
			
Self-reported previous malaria*	22	65	0.01
			
Duration of illness (days)*	5 [3-7]	3 [1-5]	0.01
			
Self administered paracetamol	95	78	0.03
			
Taken traditional remedies	39	13	0.17
			
Height (metres)	1.54 ± 0.09	1.59 ± 0.07	0.08
			
Weight (kilograms) Day 0	56.2 ± 10.4	65.3 ± 6.89	< 0.01

Data are percentage of patients, mean ± SD or median [interquartile range]

### Clinical and parasitological response

All patients in the knowlesi group responded to treatment. By symptom questionnaire, 96% reported symptomatic improvement at 24 hours. Late treatment failure was seen in one patient with vivax malaria with the presence of a rising parasitaemia on day 4 of treatment. During treatment, the maximum recorded temperature was higher in the knowlesi group than the vivax group (38.9°C versus 38.4°C respectively, *P *= 0.05), however the FCTs were similar.

Twenty six out of 33 (79%) patients in the knowlesi group and 20 of the 23 (87%) patients in the vivax group had a parasitaemia on admission that was > 1,000 parasite/μL. Assessment of parasite clearance kinetics was restricted to these patients since parasites had usually cleared before the 6 hour blood sample in those with a baseline parasite density < 1,000/μL. The admission geometric mean parasitaemia of this subgroup was 8,527 parasite/μL and 5,724 parasite/μL for the knowlesi and vivax patients, respectively.

**Figure 1:**
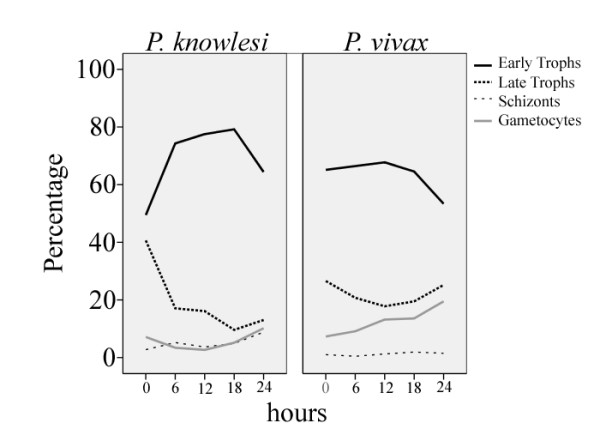
**Change in mean parasite stage distribution during the first 24 hours of treatment with oral chloroquine**.

A mixture of early and late trophozoites (mean 49.5% and 41.5% respectively) were observed on the admission blood film in the knowlesi group. However, synchronicity was observed in 19 of the 26 patients with up to 100% early trophozoites and 88% late trophozoites, while the proportion of schizonts ranged from 0 to 39%. The changing proportion of stages with time is shown in Figure 1.

**Figure 2:**
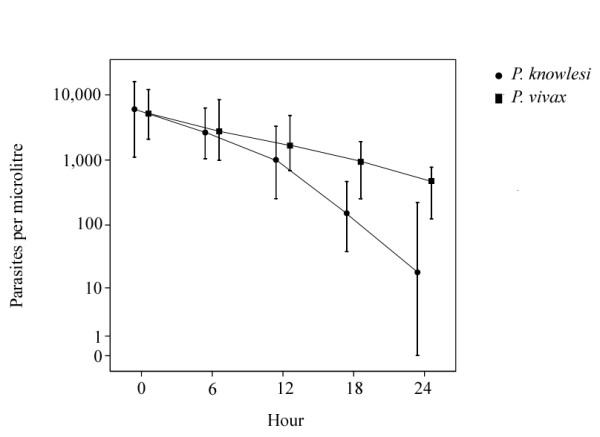
**Parasite clearance during the first 24 hours of treatment with oral chloroquine**. Graph shows median and 25%-75% interquartile ranges.

The PCT_50 _and PCT_90 _were 3.1 and 10.3 hours, respectively, in the knowlesi group (Table 2). Nine (27%) patients exhibited a rise in parasitaemia between baseline and 6 hours, but there was subsequent rapid clearance in these patients. The median calculated time to a parasite density below routine detection (< 5/μL), was 35.8 hours and 62.1 hours in the knowlesi and vivax groups, respectively (see Figure 2).

**Table 2:** Data relating to post-treatment clinical and parasitological response

Variable	*P. knowlesi *(n = 33)	*P. vivax *(n = 23)	*P *value
			
Admission parasite count (geometric mean per microlitre)	3724 (1,845-7480)	4650 (2,752-7785)	0.6
			
Fever clearance time (hours)	26.5 [16-34]	20.8 [12-32]	0.32
			
Percentage given paracetamol during hospital stay	79	52	0.05
			
Hours before fever clearance	6 [2-13]	4 [1-12]	0.48
			
Time to clear 50% of admission parasite count (hours)	3.1 (2.84-3.43)	6.3 (5.3-7.8)	0.02
			
Time to clear 90% of admission parasite count (hours)	10.3 (9.4-11.4)	20.9 (17.6-25.9)	0.02
			
Calculated time to parasite count < 5 per microlitre (hours)	35.8 [30.8-51.2]	62.1 [43.0-80.2]	< 0.01
			
Parasite reduction ratio at 24 hours	99.3 [50-100]	95.9 [58-100]	< 0.01
			
Percentage negative at 24 hours	33 (16-31)	4.6 (0-14)	
			
Parasite stages (%, range)			
Immature trophozoites	49.5 (3.1-100)	64.4 (2.7-98.9)	0.06
			
Pigmented trophozoites	41.5 (0-88.4)	28.4 (19.1-37.6)	0.09
			
Schizonts	4.0 (0-38.6)	1.9 (0-36.3)	< 0.01
			
Gametocytes	5.6 (0-32.8)	7.1 (0-53.7)	0.15
			
Slope of curve for natural logarithm parasite count			
All	0.223 ± 0.02	0.110 ± 0.02	0.02
			
Immature trophozoites	0.182 ± 0.02	0.127 ± 0.03	NT
			
Mature trophozoites	0.269 ± 0.02	0.147 ± 0.03	NT
			
Schizont	0.191 ± 0.02	0.02 ± 0.02	NT
			
Gametocyte	0.061 ± 0.01	0.012 ± 0.03	NT

Data are mean (range), mean ± SD or median [interquartile range] unless stated otherwise. NT not tested.

The parasite reduction ratio at 24 hours was significantly greater in the knowlesi group than the vivax group (99.4 [97.0-99.9]% vs 95.9 [88.6-98.4]%, *P *< 0.01). The percentages of patients with a negative parasitaemia at 24 hours were 33 (95% CI: 16-31) and 4.6 (95% CI: 0-14) for knowlesi and vivax, respectively.

To explore *P. knowlesi *dynamics, synchronous infections were selected, as defined by early or late trophozoite stages comprising > 75% of all parasite forms (knowlesi group n = 13 and 6 respectively; vivax group n = 6 and 1). There was no significant differences between early and late trophozoite predominant parasitaemias for the PCT_50 _and PCT_90 _in the knowlesi group [early trophozoites > 75% group: PCT_50 _2.1 (95% CI: 1.8-2.5) hours; PCT_90 _7.0 (95% CI: 6.1-8.28) hours. Late trophozoites > 75% group: PCT_50 _2.2 (95% CI: 1.7-3.3) hours; PCT_90 _7.3 (95% CI: 5.5-11.0) hours].

Three hourly slides performed in nine selected patients to determine stage specific clearance times found the PCT_50 _and PCT_90 _for late trophozoites to be shorter (PCT_50 _2.5 [95% CI: 2.3-2.9]) than other asexual stages, while gametocytes took longer to clear (PCT_50 _10.4 [95% CI: 9.0 - 12.2] hours; PCT_90 _34.4 [95% CI: 29.9-40.4]).

The calculated parasitaemia at the time of fever clearance was significantly lower in the knowlesi group (*P *< 0.01). The fever and parasites cleared at a similar time after treatment in knowlesi infections, while the fever cleared sooner than parasites in the vivax group.

### In vivo response

Parasite clearance, detected by nested PCR assays, took a median of 3 [interquartile range: 2-3] days in the knowlesi group. All knowlesi patients were negative for malaria by PCR on days 7, 14, 21 and 28 indicating that there was no evidence of resistance of *P. knowlesi *to chloroquine and primaquine, re-infection or recrudescence.

## Discussion

The present data demonstrate that conventional doses of chloroquine are associated with initial parasite clearance that is both faster in knowlesi malaria than vivax malaria and amongst the most rapid reported for any human malaria [13-17]. No recurrences of knowlesi parasitaemia were detected during the 28-day follow-up period, including by PCR, suggesting either a low intensity of local transmission of *P. knowlesi *or prophylactic efficacy of relatively low plasma chloroquine concentrations in the tail of the terminal elimination phase. One early treatment failure occurred in the vivax group which was likely due to the increased incidence of chloroquine-resistant *P. vivax *in Java, Indonesia, where the patient had resided until three months prior to hospital admission [18]. The exclusion of R1 resistance in the vivax group was not possible since too few patients completed follow up.

Although approximately one-third of the knowlesi patients experienced a transient rise in parasitaemia during the first 6 hours compared with only 13% of patients from the vivax group, this is likely to reflect several factors. First, the divided dose chloroquine regimen produces peak plasma concentrations that occur well after the first dose. Secondly, even if adequate plasma concentrations were reached quickly, the stage specificity of chloroquine [19] would allow late stage parasites to mature to schizogony during at least the first few hours of treatment with production of early ring forms that, as evidenced by previous own stage specific clearance times, are also relatively resistant. Thirdly, the 24 hour lifecycle of *P. knowlesi *is half that of *P. vivax *which means that, consistent with the relatively high parasite densities that are regularly observed in *P. knowlesi *infections [9], the more rapid maturation of late stage parasites would increase the likelihood of an initial rise in parasitaemia amongst the knowlesi patients. Indeed, early studies with chloroquine indicated that *P. malariae*, with an erythrocytic replication cycle of 72 hours, cleared more slowly than *P. vivax *[20]. Lastly, since most of the knowlesi patients denied a previous history of malaria, a lack of immunity might also have attenuated the effect of chloroquine on initial parasite clearance.

The study participants were aged > 15 years and lived and/or worked on tributaries of the Rejang river in close proximity to the jungle where long-tailed and pig-tailed macaques have been sighted. Despite this, previous malaria infections, and in particular recent infections, were unusual (reported by only 16 of 73 or 22% of the knowlesi malaria patients). This suggests a low level of knowlesi malaria transmission and, therefore, low background levels of immunity in the population. The successful response to treatment with chloroquine and primaquine also implies that these infections are chloroquine and primaquine naïve, supporting a zoonotic mode of transmission.

FCTs and PCTs were similar in the knowlesi group, while fever cleared before parasitaemia in the vivax group. This suggests that knowlesi malaria has a higher pyrogenic density than vivax malaria but it may also reflect differences in immune status between the two groups. Under Malaysian Ministry of Health treatment guidelines, oral primaquine was administered to knowlesi malaria patients for two days in this study as a gametocidal drug. The effect of this on both asexual and sexual forms of *P. knowlesi *is difficult to quantify. Using primaquine would seem unnecessary since gametocytes appeared sensitive to chloroquine in the first 24 hours. *P. knowlesi *is not known to have a hypnozoite stage [21]. If persistent liver stages did occur, and consistent with use of primaquine to prevent relapses of vivax and ovale malaria, longer duration treatment would be probably be necessary. Furthermore, there have been three case reports of chloroquine alone being successful in the treatment of knowlesi malaria [6,22,23].

Besides chloroquine, other anti-malarial agents have been used with apparent success for knowlesi malaria. These include mefloquine [24], quinine [10,25], atovaquone/proguanil [26] and sulphadoxine-pyrimethamine [1]. In animal models and in vitro drug studies, *P. knowlesi *is sensitive to most antimalarial drugs including clindamycin [27], naphthoquine [28], sulphonamides [29], tetracyclines [30], trimethoprim [31] and erythromycin [32]. In Rhesus macaques, a high level of innate resistance to mefloquine has been seen with the *P. knowlesi *W1 strain, while proguanil and pyrimethamine resistance were inducible in experiments with the *P. knowlesi *Nuri strain [33-35]. Although knowlesi sensitivity to artemisinin derivatives has not been reported in man, it seems very likely that it would be successful as seen in experiments in Rhesus macaques [28,36,37].

In the 1980's, oral chloroquine was thought to be more effective than oral quinine as treatment for chloroquine sensitive falciparum malaria [38]. Chloroquine was also effective and well tolerated when administered parentally or via nasogastric tube in severe falciparum malaria [39]. It may be that the use of chloroquine for severe knowlesi malaria is effective, provided that the possibility of chloroquine-resistant *P. falciparum *or *P. vivax *co-infection had been excluded. Further studies using alternative anti-malarial drugs, including artemisinin derivatives, would be desirable to define optimal management strategies for *P. knowlesi*.

## Conclusion

Chloroquine is an inexpensive, highly effective and well-tolerated anti-malarial drug for the treatment of naturally-acquired uncomplicated knowlesi malaria in humans. There is currently no evidence of drug-resistant *P. knowlesi*, reflecting a zoonotic mode of transmission and thus an absence of prior drug pressure. Further carefully-designed studies are required to determine whether chloroquine is effective in severe knowlesi malaria.

## List of abbreviations

FCT: fever clearance time; PCT: parasite clearance time; 95% CI: 95% confidence interval; PCR: polymerase chain reaction.

## Competing interests

The authors declare that they have no competing interests.

## Authors' contributions

BS and TMED designed this study and wrote the paper with CD and JC-S. CD and MZR recruited and managed the patients. CD was responsible for collection and entry of clinical and laboratory data, and together with TMED, analysed the data. SKZ and PCSD, supervised by JC-S, conducted molecular detection assays. All authors read and approved the final manuscript.
